# Building an Open Source Classifier for the Neonatal EEG Background: A Systematic Feature-Based Approach From Expert Scoring to Clinical Visualization

**DOI:** 10.3389/fnhum.2021.675154

**Published:** 2021-05-31

**Authors:** Saeed Montazeri, Elana Pinchefsky, Ilse Tse, Viviana Marchi, Jukka Kohonen, Minna Kauppila, Manu Airaksinen, Karoliina Tapani, Päivi Nevalainen, Cecil Hahn, Emily W. Y. Tam, Nathan J. Stevenson, Sampsa Vanhatalo

**Affiliations:** ^1^BABA Center, Pediatric Research Centre, Department of Clinical Neurophysiology, Children’s Hospital and HUS Diagnostic Center, Helsinki University Hospital and University of Helsinki, Helsinki, Finland; ^2^Division of Neurology, Department of Paediatrics, Sainte-Justine University Hospital Centre, University of Montreal, Montreal, QC, Canada; ^3^Department of Developmental Neuroscience, Stella Maris Scientific Institute, IRCCS Fondazione Stella Maris Foundation, Pisa, Italy; ^4^Department of Computer Science, Aalto University, Espoo, Finland; ^5^Department of Signal Processing and Acoustics, Aalto University, Espoo, Finland; ^6^Department of Paediatrics (Neurology), The Hospital for Sick Children and University of Toronto, Toronto, ON, Canada; ^7^Brain Modelling Group, QIMR Berghofer Medical Research Institute, Brisbane, QLD, Australia; ^8^Neuroscience Center, Helsinki Institute of Life Science, University of Helsinki, Helsinki, Finland

**Keywords:** neonatal EEG, EEG monitoring, neonatal intensive care unit, background classifier, support vector machine, artificial neural network, EEG trend

## Abstract

Neonatal brain monitoring in the neonatal intensive care units (NICU) requires a continuous review of the spontaneous cortical activity, i.e., the electroencephalograph (EEG) background activity. This needs development of bedside methods for an automated assessment of the EEG background activity. In this paper, we present development of the key components of a neonatal EEG background classifier, starting from the visual background scoring to classifier design, and finally to possible bedside visualization of the classifier results. A dataset with 13,200 5-minute EEG epochs (8–16 channels) from 27 infants with birth asphyxia was used for classifier training after scoring by two independent experts. We tested three classifier designs based on 98 computational features, and their performance was assessed with respect to scoring system, pre- and post-processing of labels and outputs, choice of channels, and visualization in monitor displays. The optimal solution achieved an overall classification accuracy of 97% with a range across subjects of 81–100%. We identified a set of 23 features that make the classifier highly robust to the choice of channels and missing data due to artefact rejection. Our results showed that an automated bedside classifier of EEG background is achievable, and we publish the full classifier algorithm to allow further clinical replication and validation studies.

## Introduction

Recent developments in neonatal neurological care have led to a rapid increase in the use of continuous scalp-recorded electroencephalography (EEG) for brain monitoring. Long term EEG-monitoring is now used for an individually optimized neurological treatment at neonatal intensive care units (NICU). It has been shown to be the best available method to follow cerebral recovery after birth asphyxia and other forms of brain injury, as well as the only reliable method for neonatal seizure detection (de Vries and Hellstrom-Westas, 2005; Murray et al., 2008; Boylan et al., 2013). Although neonatal EEG surveillance is becoming a standard of care for many NICUs, its 24/7 clinical review remains a global challenge (Boylan et al., 2010). To this end, clinicians have used compressed displays of the EEG activity, such as amplitude integrated EEG [aEEG (de Vries and Hellstrom-Westas, 2005)], which enables bedside visual review of EEG amplitude trends. However, the aEEG only represents one aspect of the EEG and its interpretation requires special expertise, interpretation is subjective and qualitative.

The most important challenge in the bedside EEG interpretation is how to objectively quantify temporal evolution in the spontaneous brain activity, a.k.a. “background activity” in the EEG nomenclature. This “EEG background” is known to be the most informative in assessing acute states or predicting future outcome of the brain (Monod et al., 1972; Watanabe et al., 1999; Menache et al., 2002; Murray et al., 2009). Several background scoring systems have been published over the years (Watanabe et al., 1999; Murray et al., 2009; Cherian et al., 2011), and they typically combine visually, i.e., subjectively, observed EEG characteristics to yield a holistic EEG score for an epoch that may range between scoring systems, from minutes to hours.

A number of computational EEG classification algorithms have been proposed for automated classification of newborn EEG (Stevenson et al., 2013; Matic et al., 2014; Matić et al., 2015; Ahmed et al., 2016; Raurale et al., 2019, 2020, 2021; Guo et al., 2020). They are currently expected to solve many logistic and other practical limitations in neonatal EEG interpretation by offering an objective and continuous EEG review that is harmonized across medical centres. The algorithms are generally shown to perform well compared to their clinical benchmarks, however, there is wide variability in their classifier design, the EEG classification system they have been trained on, and performance assessment. Some classifiers are based on heuristically designed computational features that are then combined using SVM-type classifiers (Stevenson et al., 2013; Matic et al., 2014; Matić et al., 2015; Ahmed et al., 2016; Raurale et al., 2019; Guo et al., 2020), while other classifiers are based on deep learning with less *a priori* crafting of the feature space (Raurale et al., 2020, 2021). Most importantly, each new study tends to advocate the latest algorithm as superior compared to the ones published earlier and overlook ambiguity in labelling of the training data resulting from inherent disagreements in the visual interpretation.

Despite the considerable development of EEG interpretation algorithms, there is strikingly sparse systematic literature on the full process that leads from visual EEG scoring to implementation of the classifier results in a clinical EEG monitor display. Here, we aimed to systematically assess the key components in developing a neonatal EEG background classifier: i) Ambiguity in the EEG background scoring; ii) the effects of classifier architectures; iii) the effects of post-processing of either experts annotations and/or classifier outputs; iv) classifier performance on individual infants, and different EEG montages; and v) possible alternatives to a clinically informative visualization of classifier outputs, using examples from unseen newborn EEG data. We chose to explore feature-based classifier designs which allow feedback on the computational EEG characteristics that are found useful for the classification.

## Materials and Methods

### Overview

**FIGURE 1:**
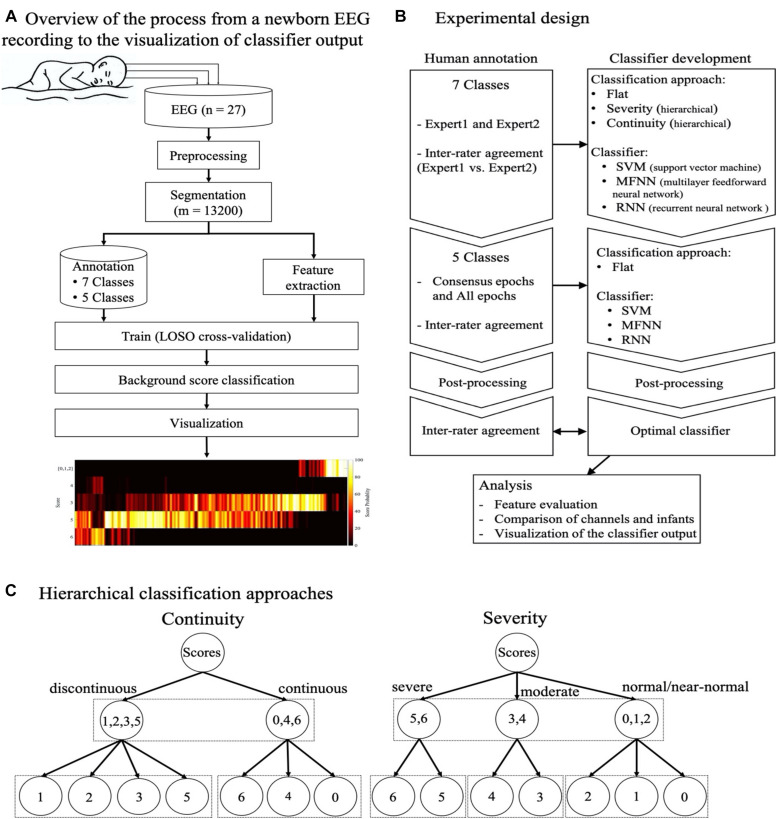
The overall design of the present study. **(A)** The flow diagram shows the steps involved from the newborn EEG recording to annotation and training of the classifier, and finally a visualization of the classifier output. **(B)** Schematic overview of classifier experiments presented in this work. **(C)** Schematic overview of two hierarchical classification approaches, one based on EEG continuity (left) and the other based on the severity of EEG background (right). As opposed the flat classifier design, the hierarchical classifiers begin with an initial distinction between continuity (left) or severity (right), followed by classifying only subset of original background classes. Abbreviations: LOSO: leave one subject out (cross-validation), SVM: support vector machine, MFNN: multilayer feed-forward neural network, RNN: recurrent neural network.

The overall design of the present study is shown in Figure 1. First, we assessed the agreement between human raters, and the potential to improve it by merging or smoothing grades. Second, we compared different classifier approaches where all background scores were either considered simultaneously (flat) or the classification was done sequentially with an initial assessing EEG continuity followed by a secondary grading procedure (hierarchical). Third, we experimented with different classifier types and training datasets. Fourth, we examined the effects of post-processing the classifier output. We then studied the performance of the proposed classifier at the level of individual EEG channels and infants. Finally, we used an independent dataset collected from a different hospital to experiment with the visualization of classifier outputs for future clinical EEG monitor display.

### EEG Recording and Processing

#### Recording

We used continuous EEG recordings gathered as a part of a prospective cohort study between 2014 and 2016 in the NICU at the Hospital for Sick Children, Toronto, Canada (Pinchefsky et al., 2019; Kamino et al., 2021). The EEG records were collected up to the first five postnatal days from 27 neonates with average postmenstrual age (PMA) of 39.7 weeks (36–41.4) at birth with clinical signs of neonatal hypoxic-ischemic encephalopathy. The duration of EEG records ranged from 8 to 102 h (average 40.5 h). The EEG was recorded using either Stellate Harmonie or Xltek Brain Monitor ICU video-EEG systems (Natus Neurology, Oakville, Ontario, Canada) at 200, 250, or 256 Hz, with 11 or 20 electrodes positioned according to the international 10–20 placement. The electrode positions used were Fp1, Fp2, F3, F4, F7, F8, C3, C4, T3, T4, T5, T6, P3, P4, O1, and O2. All EEG was de-identified before processing further for analyses.

In addition, for pilot testing of the classifier and visualization in an unseen dataset, we used four long term EEG recordings from our clinical archive of NICU brain monitoring in Helsinki Children’s Hospital. Compared to the training dataset, these additional recordings were performed by different staff in a different hospital, different recording settings (4 electrodes; F3, F4, P3, and P4), and a different EEG system (Nicolet, Natus, United States). These cases were selected based on the evolution observed in their aEEG trends, which represented typical clinical scenarios in NICU brain monitoring. The same EEG signals can be also found as example files together with the classifier algorithms in Github https://github.com/smontazeriUH/Neonatal-EEGBackground-Classifier.

#### Visual Annotation/Scoring of the EEG Background

**FIGURE 2:**
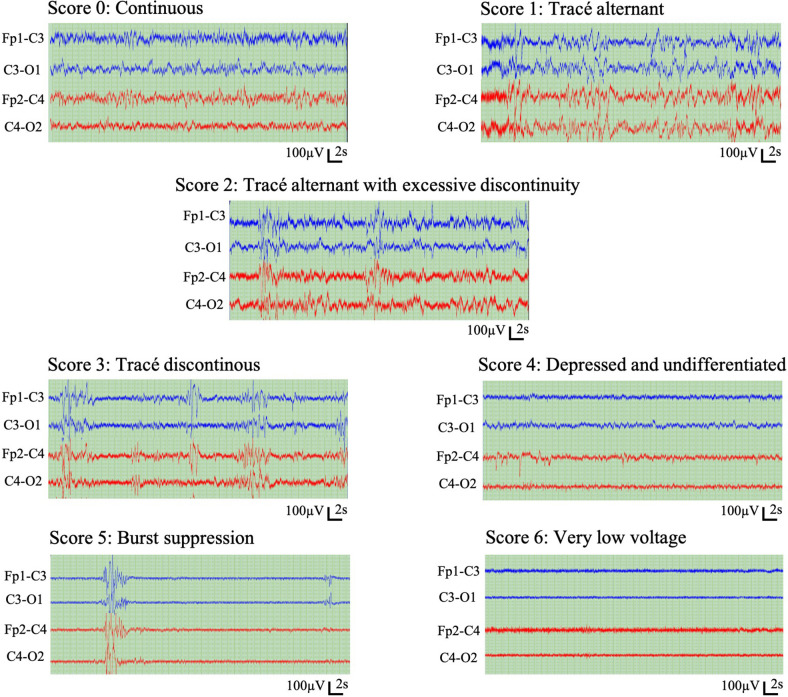
Representative example epochs of the seven scores in the visual EEG background classification.

Two expert neurologists (E.P. and V.M.; hereafter referred to as E1 and E2) annotated EEG background activity using a scoring system with seven different background scores and one score for epochs to be rejected due to excessive artefacts or seizures (Table 1). One background score was assigned to each non-overlapping 5-minute epoch, taken as the predominant activity type within that epoch, based on visual assessment of all EEG channels. The experts were fully blinded to each other’s annotation to allow the assessment of interrater agreement. Example EEG background patterns are shown in Figure 2. Scoring was performed using the montage of the expert’s own choice, which was typically a bipolar montage (Stevenson et al., 2018).

**TABLE 1:** Scoring system used in the EEG background annotation (Tsuchida et al., 2013).

Score	Description
0	Continuous
1	Tracé alternant: IBI voltage ≥ 25 μV with IBI duration ≤ 6 s
2	Tracé alternant: IBI voltage ≥ 25 μV with IBI duration > 6 s
3	Tracé discontinú: IBI voltage < 25 μV
4	Depressed and undifferentiated: persistent low-voltage background activity with amplitude between 5 and 15 μV and without normal features
5	Burst suppression: IBI voltage < 5 μV
6	Very low voltage: voltage < 5 μV or with no discernible cerebral activity

*IBI, inter-burst interval.*

The scoring system can be considered ordinal, with seven EEG background scores ordered with increasing severity: Scores 0 and 1 present the expected normal/near-normal cortical activity during active state and quiet sleep, respectively. Scores 2, 3, and 5 represent increasing abnormality in the continuity, the key feature in neonatal cortical activity, whereas scores 4 and 6 represent abnormal EEG activity with no reference to (dis)continuity. In addition, the experts were allowed to reject the epoch if they were not able to assess its background due to artefacts or excessive seizures.

Persistence of worse/higher scores during an infant’s NICU stay correlates with increasing abnormality in the long-term outcomes (Murray et al., 2009). However, this scoring system can also be perceived as a mixture of two dimensions: One dimension considers amplitude (scores 0, 4, and 6), while the other dimension considers continuity (scores 0, 1, 2, 3, and 5). These two dimensions challenge unequivocal ordering of the scores as needed in the bedside-ready visualizations later.

#### Score Merger and Smoothing

**FIGURE 3:**
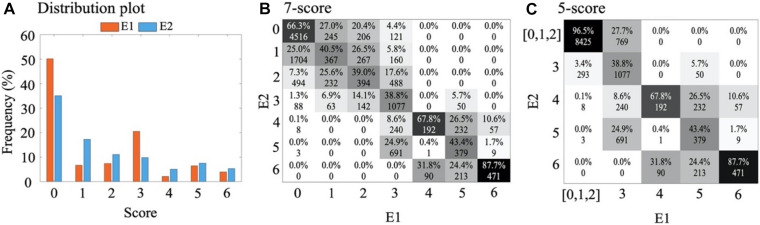
Visual annotations. **(A)** Distribution plot (in percent) of visual annotations for two experts, E1 and E2. **(B)** Confusion matrix between E1 and E2 for classification of 7-score and, **(C)** for classification of 5-score after combining scores 0, 1, and 2 into one score. The absolute values present the number of epochs corresponding to each score and the percentages of each column sum up to one.

In assessing interrater agreement (Figure 3), we found most confusion to arise between scores 0, 1, and 2. These scores are clinically less informative in the monitoring context as they characterize different vigilance states in a recovered or healthy newborn infant. Hence, they were merged to yield five scores. In addition, we applied temporal smoothing on annotations to remove noise (Supplementary Figure 2D) in the annotation time series. To this end, we tested different lengths of smoothing window from 3 to 13 epochs and found a window with five epochs (25 min) to give the best classifier performance.

#### Merging Expert Annotations for a Combined Classifier

The final classifier model was trained using scores from both experts to include information from both their consensus as well as disagreements. To this end, we trained each classifier using either consensus epochs (CONS) and all the epochs (ALL). The second approach forces the classifier to use all available information, giving more weight to consensus epochs while also retaining score information from epochs with disagreement between experts. To evaluate classifier performance, we evaluated the models against scores from consensus epochs as well as scores from each expert separately.

#### Pre-processing

The continuous EEG signal was pre-processed with an automated pipeline. First, all the EEG channels were scanned visually to find and discard very poor quality signals (e.g., detached electrodes), followed by automated scanning for high-amplitude values or flat signals (constant value). All samples exceeding an amplitude of ± 500 μV were detected as artefacts. Third, each EEG signal was band-pass filtered at 0.5–35 Hz with a 5th order Chebyshev Type II filter. Fourth, signals were resampled to 64 Hz with an anti-aliasing filter, and segmented into 5-min long non-overlapping epochs (discrete signal length of 19,200 samples) corresponding to the visual annotations. Fifth, we rejected a channel in an epoch if at least 25% of the signal in the given channel was detected as artefacts (0.32%; *N* = 715). We rejected the whole epoch if at least 50% of the channels were rejected (0.24%; *N* = 30).

#### Feature Extraction

All features were computed initially for the referential derivations, and features for additional bipolar derivations (F3-P3, F4-P4, and P3-P4) were computed for montage comparisons. We computed altogether 98 computational features for each derivation and epoch of EEG data to capture aspects of amplitude, complexity and oscillatory behaviour. The feature set was gathered from a large number of previous publications and implemented into Matlab locally in our laboratory. For a full list of features, their classification and literature references, please see Supplementary Table 3. All feature algorithms can be found in Github^1^. Features were initially calculated on each channel within a 5-min epoch and then summarised across channels using the median.

### Classifier Designs and Training

#### Overview

Three classifier designs were trained and assessed, all of them using the same computational features. The ultimate “proposed classifier” is also published in Github (see text footnote 1). The classifiers were based on (i) support vector machine (SVM), (ii) multilayer feedforward neural network (MFNN) or (iii) recurrent neural network (RNN). The first two do not consider linkages over consecutive epochs, while the last one captures long-term dependencies in signal (or annotation) sequences. Intuitively, it is reasonable to expect that some level of temporal correlation exists in neonatal EEG that is characterized by gradual evolution rather than abrupt changes of EEG states. The common clinical practice of EEG scoring is also trained to favour smooth changes in background scores, which implicitly introduces temporal correlation to the score sequences. In addition, we tested whether merging annotations to a smaller number of scores would improve classifier performance. In all classifier builds, the input features were normalized at each training iteration to z-scores. Classification performance was assessed using leave-one-subject-out (LOSO) cross-validation which splits the data into train and test sets based on the subjects without any overlap between training and testing data. In LOSO cross-validation each fold is one subject’s data.

#### SVM Classifier

The SVM was implemented using the *fitcecoc* function in Matlab (version R2019a). This function trains the multiclass error-correcting output codes (ECOC) model using *K*(*K*−1)/2 binary SVMs. These binary SVMs use the one-vs-one coding design, where *K* is the number of unique class labels. We used linear kernel for SVMs since in initial testing we found no significant improvement for other kernels over the linear kernel. Hyperparameters of the SVM were optimized by Bayesian optimization within an internal fivefold cross-validation.

#### MFNN Classifier

A multilayer feed-forward neural network (MFNN) was developed with linear input layer and two hidden layers and output layer with hyperbolic tangent sigmoid transfer functions (Supplementary Figure 1). The neurons in adjacent layers are fully connected with weights and biases while the neurons in the same layer are not interlinked. The optimum number of hidden nodes is very difficult to determine and needs extensive experience. In this work, it was found that by choosing the number of hidden nodes as shown in Supplementary Figure 1, accurate results could be obtained. Classification output for each input sample corresponds to the maximum valued label after using the *softmax* function of the output of the MFNN.

Backpropagation learning (BP) with the stochastic gradient descent algorithm was used for the supervised training process of the MFNN. In order to prevent the classifier from overfitting, we kept 10% of the data in the train folds of LOSO aside from training to use as an inner validation set. Weights were updated in batch mode with a dynamic set of learning rate (LR) and a constant momentum factor (MF). Employing MF would help the training procedure escape local minima and reduce the likelihood of instability (Basheer and Hajmeer, 2000). LR value at the first iteration of the training was set to 0.1 and after a set of 6,000 training iterations, it was scheduled to be reduced by 75%. MF value was set to a constant value of 0.8. The training process terminated after 120 000 iterations or if the Root Mean Square Error (RMSE) on the training pattern was less than 1 × 10^–3^.

#### RNN Classifier

The RNN model was developed based on the proposed MFNN. The second hidden layer of the proposed MFNN is connected to one feed-backward layer characterized by one step time delay as demonstrated in Supplementary Figure 1. The idea of using RNN was to consider information lying in the sequence of epochs such that the previous epoch provides useful information for the classification of the current epoch. Real-Time Recurrent Learning (RTRL) was used for supervised training of the RNN (Williams and Zipser, 1989). In RTRL learning, the weights at iteration *K* are modified by the errors back propagated from iteration *K+1* through the recurrent layer. Weights in the forward path of RNN initialized to the weights from trained MFNN classifiers (used as pre-trained weight initialization) and weights in the backward path initialized to small random numbers. During the training, all the layers fine-tuned with a very small LR set for each unique class of output independently based on the distribution of that class.

#### Classification Approaches

We tested both flat and hierarchical classification approaches. In a flat classification approach, no inherent hierarchy between the scores is considered and the output relies on a single decision of the classifier including all the scores. The flat classification was tested with all the three types of classifiers (SVM, MFNN, and RNN). In a hierarchical approach, in turn, the seven initial EEG scores are grouped into three new scores based on their phenomenological properties or clinical inference. To this end, we used two strategies illustrated in Figure 1C. The first strategy was grouping the scores according to “continuity” of the signal, and the second strategy was grouping the scores according to the clinical inference of “severity” of the score (normal, moderate, and severe). Using hierarchical approaches disrupts the sequence of epochs while the RNN requires ordered sequential inputs. Therefore, hierarchical approaches are only applied to SVM and MFNN classifiers and only a flat classification approach is implemented for RNN. We also used the Synthetic Minority Oversampling Technique (SMOTE), with *k = 10*, to compensate for the imbalanced distribution of scores (Chawla et al., 2002).

### Post-processing and Feature Evaluation

Classifier performance can be improved significantly by postprocessing (Ansari et al., 2016). A random misclassification, seen as single epoch spikes in the score time series (Supplementary Figure 2D) could be tempered by applying a temporal smoothing that aims to emulate the relatively smooth (tens of minutes to hours) state transitions in the real-world situations.

Features were evaluated using feature selection methods to find out their relative impact of each feature for classification accuracy. We scanned through four different artefact thresholds (0, 10, 25, and 50%) to also find out whether features are differentially sensitive to increasing level of artefacts in the EEG data. The Genetic Algorithm was utilized in a wrapper approach to search for the optimal feature subset (John et al., 1994). This algorithm is considered effective and powerful global search tool for finding feature subsets from large-scale and poorly understood feature spaces (Siedlecki and Sklansky, 1993; Emmanouilidis et al., 2000; Kudo and Sklansky, 2000; Oliveira et al., 2003). The wrapper approach operates in the context of the learning model and involves the computational overhead because of evaluating each candidate feature subsets by model. In our analysis, each feature selection searched between 5,000 combinations of different feature subsets, where every subset has different numbers of features. The following settings were applied for the feature selection: (1) population size is 100 sets each containing different numbers of features. Thus, each feature can be selected a maximum of 100 times; (2) two-point crossover rate is 0.6; (3) mutation rate is 0.1; (4) stopping condition is 50 generations.

#### Performance Measures and Statistical Analysis

The performance of the classification was assessed using percentage of accuracy and F1-score metrics for macro and weighted averages (M-Acc, M-F1 score, W-Acc, and W-F1 score), across all infants (LOSO-folds). The 95% confidence interval (CI_95%_) from bootstrap resampling (n_*resampling*_ = 1,000) was used to declare the statistical significance in accuracy and Cohen’s κ between classifiers.

The output of each trained classifier was compared to the annotations of the human experts by measuring inter-rater agreement using Cohen’s κ. The classifiers trained on the smoothed combined scores which outperform other strategies were compared to the annotations of the human experts by measuring the pairwise κ. The overall agreement between human annotation (E1 and E2) and a composite of a human expert and a classifier was evaluated to determine whether the predicted scores are non-inferior to the human expert considering the subjectivity of human annotation.

## Results

### Visual Annotations and Interrater Agreements

The final annotation dataset from both experts consisted of 13,200 epochs (approximately 1,100 h) of EEG. As expected, the distribution of scores was somewhat skewed toward the lower value scores that indicate better brain function (Figure 3A). The number of epochs rejected as ungradable by E1 and E2 were 384 and 1,313, respectively.

**FIGURE 4:**
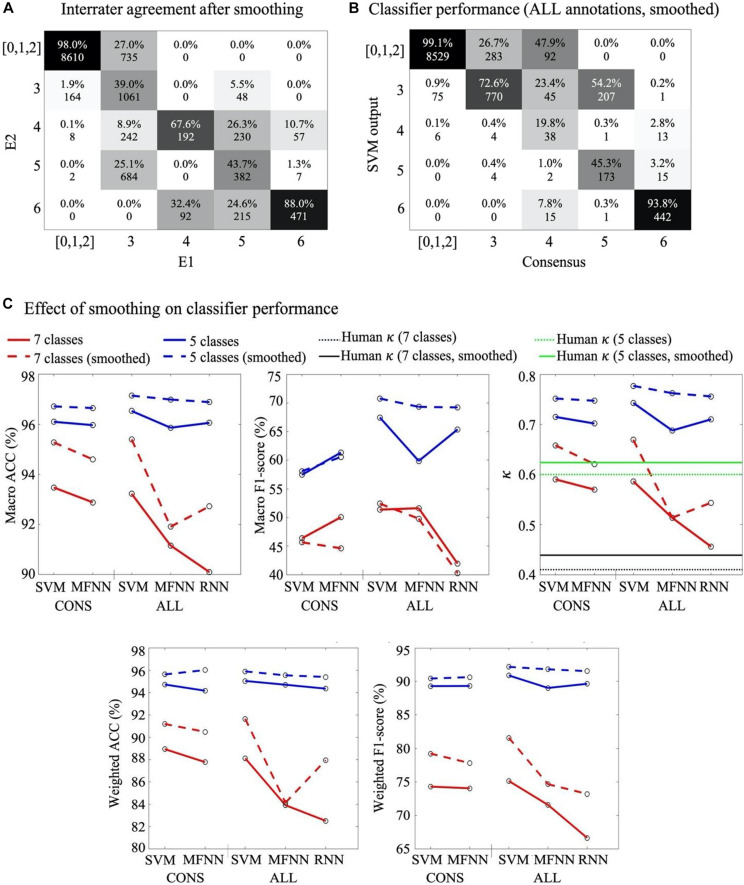
Effects of post-processing. **(A)** Confusion matrix between experts 5-score scores after smoothing. Note the significant increase in agreement as compared to Figure 3. **(B)** Confusion matrix for SVM classifier from ALL annotation approach based on 5-score with smoothing. **(C)** Comparison of classifiers’ performances without (solid line) and after with (stippled line) smoothing. Note the generally higher performance after smoothing. In the right most graph, the horizontal lines depict comparison to the human interrater agreement levels using 7-score (κ = 0.41; black stippled line), smoothed 7-score (κ = 0.44; black solid line), 5-score (κ = 0.60; green stippled line), and smoothed 5-score (κ = 0.62; green solid line), respectively.

The inter-rater agreement using all seven background scores was moderate (κ = 0.41). A closer inspection of the confusion matrix Figure 3B between E1 and E2 shows two clusters of disagreements: there was more overlap within the group of scores referring to normal or near normal EEG (scores 0–2) as well as the group of scores referring to more severe EEG (scores 3–6). On top of that, there was also an overall tendency of one expert (E2) to assign lower background scores compared to the other expert (*p* < 0.001, paired *t*-test). This suggests ambiguity among neighbouring scores, which could be handled by merging such scores for a better classification performance. To this end, we considered the clinical interpretation of each subscore: Since the normal/subnormal scores (0, 1, and 2) all have the same overall clinical interpretation of favourable recovery (Murray et al., 2009), we decided to create an alternative 5-score system by merging them into one score. Indeed, the new 5-score scoring gave a considerably higher interrater agreement of κ = 0.60. In the following, we therefore decided to train and test each classifier for both the original 7-score and the revised 5-score. In addition, the annotations were smoothed with a moving window of five epochs (Supplementary Figure 2D for an example) which increases the inter-rater agreement (κ = 0.60 to 0.62) (Figure 4A).

### Optimal Classifier

#### Flat vs Hierarchical Classification Approaches

The results comparing different classifier approaches are detailed in Supplementary Table 1. Results show no significant differences in performance between the flat approach and any of the hierarchical approaches (bootstrap average class accuracy: SVM–Flat CI_95%_: 87.1–87.6; Continuity CI_95%_: 86.8–87.2; Severity CI_95%_: 86.4–87.1). We, therefore, decided to only use the flat approach in further experiments as it is less computationally intensive.

#### Merging Expert Annotations Into a Unified Classifier

We trained and tested classifiers by using either consensus epochs alone (*CONS*), or by using all annotations (*ALL*). The latter was accomplished by training the classifier twice, using both sets of annotations, hence considering disagreement labels as well, but giving twice the weight to consensus labels. The first approach limits the dataset to epochs with consensus only (total *N* = 7396; on average 7,122 in each training fold), and consequently disrupts the sequence of epochs, precluding use of RNN classifiers.

In general, the classifier performance was higher against consensus annotations, irrespective of whether the classifier was initially trained using *CONS* annotations *vs ALL* annotations (Supplementary Figure 2). The performance of SVM and MFNN classifiers was mostly comparable, however, MFNN performed better in terms of macro f1-score. The RNN classifier was generally poorer, which is likely due to the sensitivity of ANNs to data conflicts (Versaggi, 1995; Raggad, 1996) in the multi-rater annotations (Supplementary Figure 2).

#### Post-processing With Score-Merger and Smoothing

The confusion matrices of both expert annotations (Figure 3) and classifier outputs (Supplementary Figure 2) suggest that the majority of ambiguity arises from neighbouring scores. In particular, there are also clinical reasons to combine at least the first three (0,1,2) scores with shared clinical information value in the monitoring context. To this end, we measured changes in classification accuracy from adopting a 5-score scoring system (*m* = 13200 available epochs, Figure 3). Comparison of a range of smoothing windows indicated the best classifier performance when using median smoothing with a window of seven consecutive epochs (Figure 4). The overall performance of all classifiers improved considerably when reducing the number of scores and applying temporal smoothing.

#### The Proposed Optimal Classifier

The results from the SVM, MFNN and RNN classifiers trained on *ALL* annotations were mostly comparable, and generally better than classifiers trained with *CONS* annotations (almost 10% in terms of f1-score, Figure 4 and Supplementary Table 2). We found the best performance with a SVM classifier trained on *ALL* annotations, 25% threshold for excluding epochs, a 5-score classification system with temporal smoothing on both the input annotations and classifier output.

### Classifier Output vs Human Inter-Rater Agreement

In principle, classifier performance should not be less than the degree of inter-rater agreement between human expert annotations. Human interpretation is bound to be imperfect, due to possible inconsistencies in the human perception, or due to genuine ambiguities in the signal itself with respect to the given classification task. In the absence of an absolute ground truth in the EEG scores, it may be useful to benchmark the classifier also to the level of human performance (inter-rater agreement). As shown in Figure 4C, the agreement between the best performing classifiers and the human experts were higher than the agreement between human experts.

### Feature Evaluation

**FIGURE 5:**
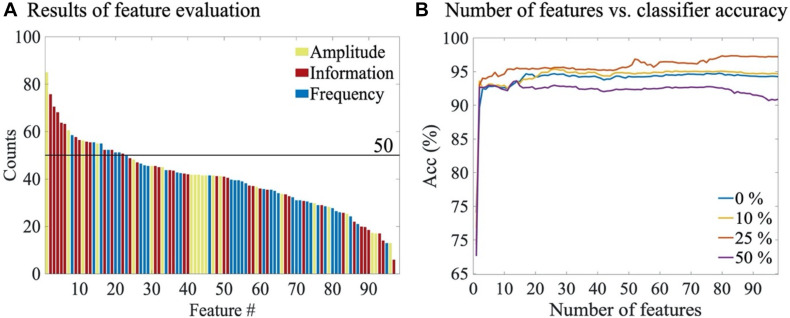
Feature selection results. **(A)** The average number of times each feature was selected through all artefact rejection thresholds. Colouring refers to the feature categories **(B)** The overall accuracy of SVM classifiers as the number of features is increased (also Supplementary Table 3). Results are shown for different artefact rejection thresholds.

Feature selection was carried out using the unified SVM classifier trained on *ALL* annotations. Comparison of four different artefact rejection thresholds (i.e., 0, 10, 25, and 50%) showed that artefacts do affect the features selected for an optimal classifier: The average number of features selected with the artefact thresholds of 0, 10, 25, and 50% were 37 (IQR: 16–62), 31 (IQR: 15–54), 50 (IQR: 18–83) and 51 (IQR: 14–83), respectively. Importantly, 23 out of 98 features were robust enough to survive in over half of the times through all artefact rejection thresholds; this suggests that a set of heuristic features are highly robust to common NICU artefacts in the EEG data (Figure 5A). When adding features to the model one at a time, there was a clear ceiling effect after only a few features (Figure 5B), and the rejection threshold 25% appeared to provide consistently best classifier performance. The top five features with 25% rejection threshold were: the standard deviation of the amplitude modulation, activation synchrony index, the average of the amplitude modulation, the average slope of the multiscale entropy curve, and power in 9–11 Hz sub-band. Notably, the overall classifier performance tended to deteriorate when more than three thirds of the feature set were included. See Supplementary Table 3 for detailed results of the feature selection process over all the four thresholds.

### Classifier Performance in Individual Channels and Infants

**FIGURE 6:**
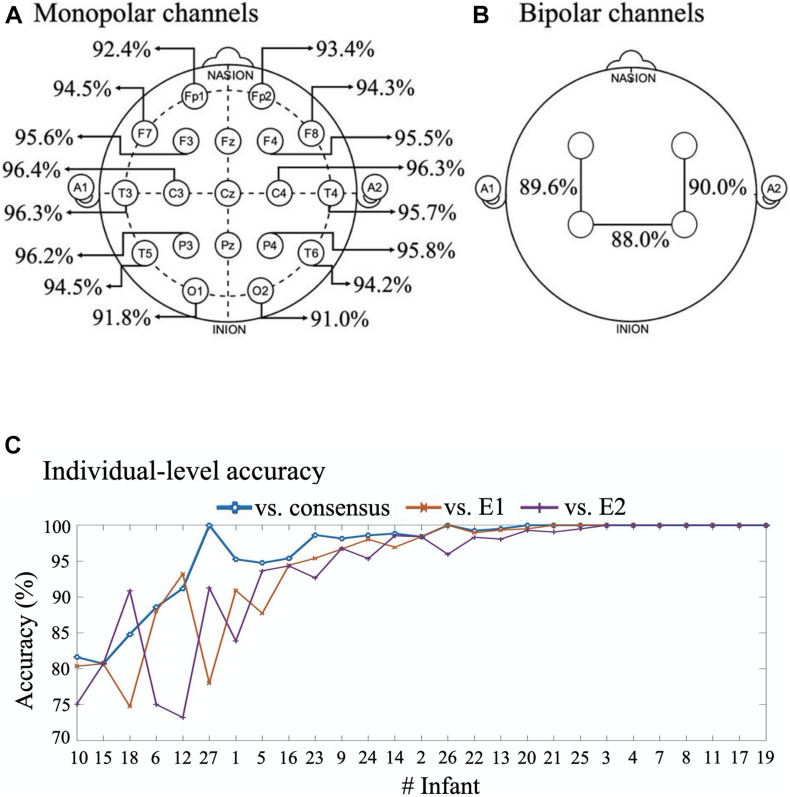
Comparison of classifier accuracies in all channels and individuals. **(A)** 16 channels with monopolar montages. **(B)** Bipolar montages. Where in six infants with only 8-channel recording these montages are Fp1-C3, Fp2-C4 and C3-C3, and for 21 infants with 16-channel recording these montages are F3-P3, F4-P4, P3-P4. **(C)** Per infant classification accuracy vs consensus, E1 and E2 annotations. The infants are here ordered according to their mean classifier accuracy. All performance measures in this figure are given for the optimal classifier.

Comparison of classifier performances between individual electrodes showed that there is spatial variation in accuracy (Figure 6). However, the differences are relatively minor, which is likely due to the significant contribution of the common reference electrode, as well as the global nature of background activity. We then assessed the performance of bipolar derivations that are typically recorded in the few channel aEEG monitoring. All three bipolar derivations yielded a mutually comparable classification accuracy of 88–90% (Figure 6B), which was only a few percent lower compared to the monopolar recordings (Figure 6A). The slightly lower performance of bipolar derivations is perhaps caused by the training of the algorithm using monopolar signals. Notably, the even performance across channels and derivations suggests that an algorithm could achieve a very high accuracy even when reducing the number of recording electrodes down to one electrode with sufficient signal quality.

As clinical work involves treating single individuals, it was essential to evaluate the classifier performance at the level of individual infants (Figure 6C). When comparing the classifier accuracy to the EEG epochs with consensus scoring, the accuracy ranged from about 80 to 100%. A little more variance was observed when comparing classifier to each expert alone, with accuracy ranging from about 74 to 100%. The classifier accuracy was above 90% in two thirds of the infants.

### Visualization of the Classifier Output

An essential part of bedside implementation is to visualize the classifier output in an intuitive and transparent manner. Ideally, the visualization needs both the classifier output, and an estimate of its certainty to inform the clinician of, e.g., ambiguity in the EEG signal for biological or technical reasons. To this end, we visualized classifier outputs in two different ways: a heatmap output and background trend output. In the heatmap output, colours from “hot” colormap are used to depict probability (i.e., posterior in the SVM classifier) of each score. In a background trend (BT) output, a continuous signal is estimated by taking a weighted average of probability values. A moving average filter with window length of three epochs is then used to smooth the estimated signal. The output uncertainty is demonstrated in this visualisation with highlighted areas around the BT line.

**FIGURE 7:**
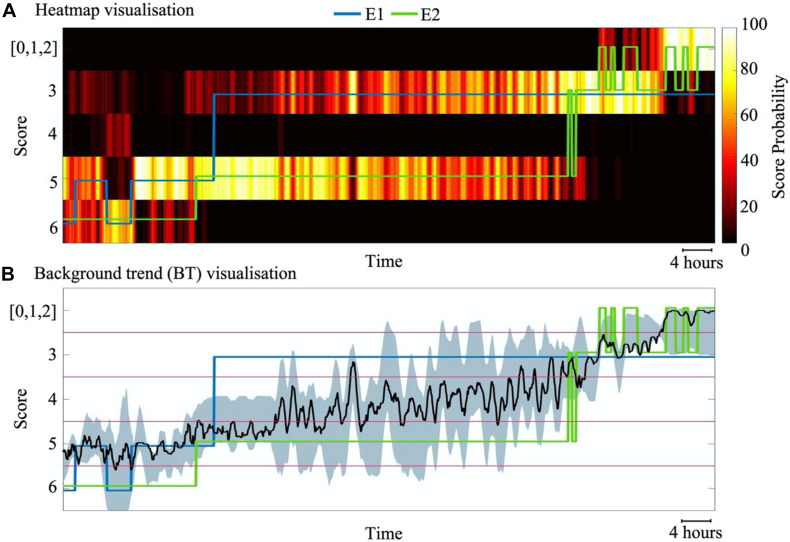
Visualization of SVM classifier outputs for an example subject with 88 h of data. **(A)** A heatmap visualisation that depicts the time course of normalized probability for each score. **(B)** Visualization of a background trend (BT) shows the weighted average of probability values (black line) as well as the uncertainty of the classification at each epoch (grey shade). The blue and green lines indicate the individual annotations from experts E1 and E2, respectively.

As shown in Figure 7, there may be substantial periods when the EEG falls between neighbouring classes in both the automated classifier assessment and the scorings by different experts. In addition, we noted in many cases that the classifier tends to consider score 4 as an extension of the normal/near-normal background (scores 0, 1, and 2) rather than a continuum between scores 3 and 5.

**FIGURE 8:**
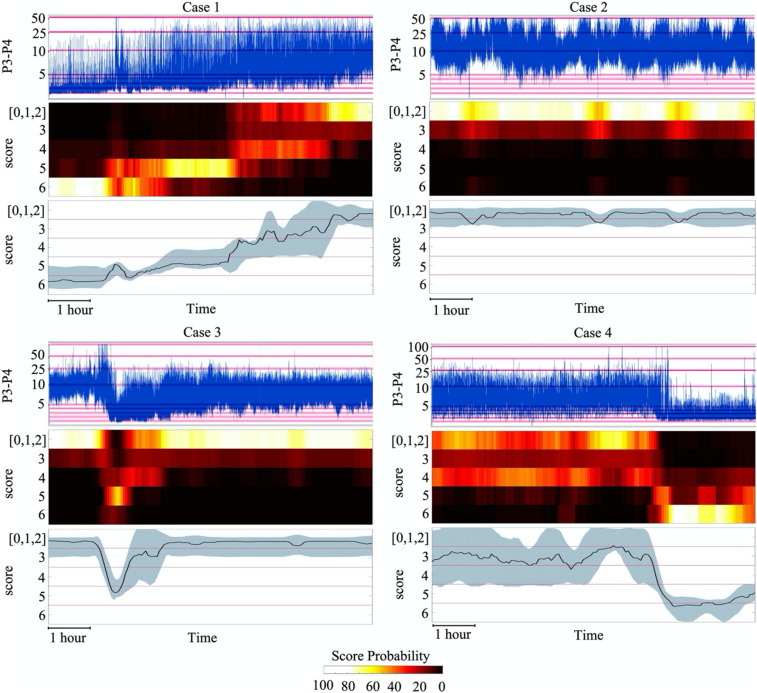
Visualization of SVM classifier outputs and its comparison to the aEEG trend display using an unseen EEG monitoring data. The colours in the heatmap visualisation indicate the probability of the given score (normalized over all scores at each 5 min epoch). Note the overall match in the temporal evolution of brain activity seen in both the aEEG trend and the classifier outputs. In BT visualisation, the classifier output is converted to a unidimensional score that depicts the likeliest score and its confidence, showing how the real evolution of brain activity goes through non-discrete transition toward better scores.

Finally, we carried out a proof of concept testing of classifier generalization and result visualization by using a set of four infant recordings from a different medical centre, recorded with a different EEG system, as well as different electrode types and configurations. The EEG background evolution in the classifier output was visually compared to the concurrent evolution of aEEG patterns to provide the clinically most transparent and intuitively understandable assessment. As shown in Figure 8, the classifier output depicts graded evolutions in cortical activity, which are closely in line with the traditional clinician reading of aEEG trends. At the same time, however, the classifier often tends to suggest that score 4 is closer to score 0–2 than somewhere between scores 3 and 5.

The first infant (case 1) shows a typical evolution after birth asphyxia, starting from an essentially inactive EEG which is gradually and smoothly replaced by recovering, better forms of background activity. The longer period of burst suppression is clearly seen in the classifier output, however, it is not seen in the aEEG trend due to the very commonly seen (Toet and Lemmers, 2009; Marics et al., 2013) artefactual elevation of the lower aEEG boundary. The fourth quarter of the figure shows a relatively low amplitude non-fluctuating activity in the aEEG, which is nicely depicted in the classifier output of grade 3–4, with a wider uncertainty around it.

The second infant (case 2) is a typical case seen after appropriate recovery or milder incidents. It shows mostly normal/near-normal background pattern that fluctuates between fully continuous and varying levels of discontinuity. The classifier output suggests most strongly the highest score, with a low probability of the increased intermittency. This information is beyond the reading that can be seen from the aEEG trend which only suggests a fluctuating [a.k.a. cycling (Osredkar et al., 2005; Thoresen et al., 2010)] activity within normal amplitudes.

The third infant (case 3) shows a sudden drop in cortical activity, which was here for an unknown cause, but it can be due to a range of medical incidents (e.g., medication, hypoglycemia, hypoxia, cerebral bleeding) (Olischar et al., 2004; Ter Horst et al., 2004; Weeke et al., 2017). The EEG activity exhibited genuine burst suppression for a brief time as also reflected in the aEEG trend. This was rapidly replaced by low amplitude activity (score 4), and then by several hours of nearly normal EEG pattern with somewhat increased discontinuity (shown with the lower probability of score 3). Notably, the aEEG trend reflects this background evolution generally well; However, aEEG trend does not disclose the prolonged increased discontinuity that has predictive clinical value (Watanabe et al., 1999; Murray et al., 2009).

The fourth infant (case 4) exhibits aEEG activity that appears to be somewhat lower than typical, until it suddenly drops to nearly inactive for an unknown cause. Until suppression, the classifier depicts some level of probability for multiple classes, especially for score 0–2 and score 4. The BT line shows wider uncertainty and a mean value near score 3. While this indicates that EEG activity is in the border zone, it also demonstrates a case where two-dimensional scoring system (amplitude vs continuity; see methods for discussion) results in problems with one-dimensional visualization.

## Discussion

Our findings show that automated classification of neonatal EEG background activity is possible with high accuracy, even at the level of individual infants. The process of developing such classifier algorithms involves a longer chain of steps, starting from the bedside EEG recordings until the classifier is implemented in the bedside monitoring display. Our results are generally consistent with prior studies showing that automated classification of neonatal EEG background is possible with reasonable level of accuracy (Stevenson et al., 2013; Matic et al., 2014; Matić et al., 2015; Ahmed et al., 2016; Raurale et al., 2019, 2020, 2021; Guo et al., 2020). We extend the prior literature by systematically characterizing the steps in classifier development, and describing the impact of choices made by clinicians and engineers at key steps of the process. While modern development of machine learning algorithms has become by and large a “black box” exercise (Chen et al., 2019; Roy et al., 2019; Borjali et al., 2020), developing tools for future evidence based medicine would call for more transparency. For example, exposing the bottlenecks, sensitivities, ambiguities and points with robustness will greatly benefit future development of automated methods in EEG analyses. Our choice to use feature-based methods rather than deep learning methods (e.g., CNN) was motivated by the need to learn of the weakest links in the process chain, before and after the technical classifier solution.

### Comparison With Prior Literature

The estimated accuracy of our classifier is comparable to other neonatal EEG background classifiers (Stevenson et al., 2013; Matic et al., 2014; Matić et al., 2015; Ahmed et al., 2016; Raurale et al., 2019, 2020, 2021; Guo et al., 2020). Direct comparison of classifier performances is severely compromised. Firstly, the EEG scoring system is variable between laboratories and datasets, both with respect to the number of classes as well as their conceptual content. For instance, the scores are typically characterized by a varying mixture of descriptions referring to EEG continuity, amplitude, or longer temporal structure such as sleep-wake cyclicity. This variability makes it impossible to even convert *post hoc* one scoring system to another by a straightforward score merger or split. Secondly, prior literature has used widely varying epoch lengths to score the EEG. Our work used 5 min epoch to resolve changes in brain state in a clinically relevant time span of tens of minutes (Figure 8). Thirdly, classifiers may also be trained using different numbers of EEG signals, ranging from classifiers based on single EEG signals/channels (Raurale et al., 2020, 2021) to solutions that require a larger number of concurrent EEG signals (Stevenson et al., 2013). Since EEG background is by definition referring to a global brain state, using multiple EEG channels is theoretically redundant though it may bring practical benefits in classifier robustness as neonatal EEG is commonly contaminated with a variety of artefacts. The comparison of individual channels and the bipolar derivations of routine neonatal aEEG monitoring (Toet and Lemmers, 2009; Thoresen et al., 2010; Tsuchida et al., 2013) is crucial for assessing the potential for clinical implementation. Together with some prior studies (Ansari et al., 2020), our findings suggested that a background classifier can be stable enough at single channel level, and robust enough to variation in signals across cortical areas. Moreover, we show here that the classification accuracy can be high at the level of individual infants, which is a requisite for clinical utility.

### Clinical Background Score Is the Bottleneck in Classifier Development

The most significant bottleneck in developing machine learning based neonatal EEG classifier is the lack of firm ground truth for solid training. All clinical scoring systems are phenomenological, they give verbally explicit descriptions that are subjectively applied, score boundaries are arbitrary and often inherently ambiguous and not necessarily physiologically meaningful. This has led to a heterogeneous clinical and classifier literature. At best, it may be reasonable to assume that the worst and the best background scores accurately reflect the ends of the same spectrum; However, scores in the middle consist of phenomena in different dimensions, such as signal amplitude, continuity or long-term temporal structure (sleep-wake cycling). We adopted a scoring system with seven classes as described by Tsuchida et al. (2013), which includes different classes for amplitude (scores 0,4,6) and continuity (scores 1,2,3,5). We tested a hierarchical classifier to initially distinguish between continuities before a final label is assigned, however, this did not improve the overall classifier results.

The long held clinical neurophysiology tradition of treating amplitude and continuity separately (Murray et al., 2009; Tsuchida et al., 2013) is challenged by the bedside practise where changes in brain state after e.g., recovery from birth asphyxia are observed as unidimensional change in background activity from inactive to normal (Toet and Lemmers, 2009; Thoresen et al., 2010; Tsuchida et al., 2013). Some other background scores are more designed for such one-dimensional assessment (Murray et al., 2009; Korotchikova et al., 2011; Stevenson et al., 2013). However, they may include sleep-wake cycling which needs tens of minutes to hours per epoch which decreases the temporal resolution needed in practical bedside brain monitoring.

In addition, score boundaries are arbitrary as they are based on consensus statements rather than generated from data or underlying physiology. Therefore, it comes perhaps as no surprise that multiple human experts have substantial level of disagreement, especially between neighbouring categories. Assessing inter-rater agreement has become popular with expansion of EEG monitoring practises (Dereymaeker et al., 2017; Wusthoff et al., 2017; Massey et al., 2019; Stevenson et al., 2020). However, most works on classifier design have used consensus scores which by design eliminates ambiguity in individual annotations, data or scoring system. Here we exploited this latent human insight by training the classifier with independent expert scores. It is surprising in this context how little attention has been given to how to disambiguate the EEG scoring system in the first place? Or how to generate clinically appropriate, unidimensional score categories to allow easier visualization of classifier outputs (for an example, see Figure 8), akin to indices used in NIRS or vital signs monitoring. An ideal scoring system should be designed so that it strikes an optimal balance between maximizing inter-rater agreement and value of clinical information of each ordinal category. To this end, it is crucial to uncover the natural structure in the neonatal EEG signal beyond visually identified grapho-elements. Recent advancements in self-supervised learning methods (Banville et al., 2019, 2020) hold promise for a genuine bidirectional dialogue between machine learning and clinical neurophysiology.

### Feature-Based vs Deep Learning Methods in EEG Classification

Here we used SVM, MFNN and RNN classifiers and three training approaches. While many recent studies have developed end-to-end deep learning approaches, we chose to explore the feature-based approach which allows a more transparent assessment of the process. A feature-based system can also be easier to train with limited and technically variable datasets. Here we show that a feature-based method can perform at high enough accuracy for clinical implementation, and it is surprisingly robust to artefacts and missing data. Our work shows that optimal classifier design requires further development of the clinical background scoring system for less ambiguity, after which it will be useful to examine the added value of deep learning methods compared to feature-based method. An essential further factor with deep learning is the shortage of labelled data, which may be overcome by employing modern data-driven methods for self-supervised feature extraction of natural EEG signal properties (Banville et al., 2019, 2020).

### Visualization of Results for Clinical Implementation

A bedside implementation of classifier outputs needs visualization that is transparent, accurate and clinically informative. Some visualizations have been attempted in the past based on a combination of binary and probabilistic trace of discrete score categories (Temko et al., 2015). Here, we developed this idea further by demonstrating how the probability of discrete score categories could be visualized using heatmaps, which often gives time-varying likelihood for multiple background scores. While this is perhaps the most comprehensive display, it may also be confounding for a bedside clinician who would still need to integrate multiple levels of information for conclusions. As an alternative, we also probed the idea of using a unidimensional background score which better corresponds to the display used in vital signs or NIRS monitoring. This visualization is far easier to interpret and it also allows the addition of a confidence measure which maybe essential for the bedside clinician.

### Future Directions

All automated EEG assessment may benefit from improving the pre-processing stage before feature extraction/classification. Our analytic pipeline includes a crude, automated artefact recognition, however, more sophisticated artefact recognition methods (Stevenson et al., 2014; Kauppila et al., 2017) could improve classifier performance. In addition, the incorporation of automated seizure detection could help recognize epochs where excessive seizure activity interferes with the EEG background.

Finally, future clinical studies will be needed after implementing the classifier into a functioning EEG monitor. In order facilitate further development, replication and validation, we have shared the full classifier algorithms (from the feature extraction to result visualization). The final clinical validation of the system will consist of assessing its conformance to clinical assessment at individual level, as well as evaluating the clinician’s perceived added value of the automated EEG background classifier for bedside decision making.

## Data Availability Statement

The datasets presented in this article are not readily available because they were provided by a third party (Hospital for Sick Children, Toronto, ON, Canada). Requests to access the datasets should be directed to (elana.pinchefsky@mail.mcgill.ca).

## Author Contributions

ET, EP, and CH performed the patient recruitment, EEG collection, and the design of the visual scoring system. EP and VM performed the visual classification. SM, MK, KT, and NS carried out the computational EEG data analysis (incl. features). SM, NS, IT, JK, MA, and SV developed the incremental classifier designs. SM, NS, and SV developed the overall manuscript content. SM, NS, and SV prepared the manuscript and figures, while all authors carefully reviewed, and commented and finally approved the article. ET, CH, NS, and SV arranged the funding and each for a different aspect of the work.

## Conflict of Interest

The authors declare that the research was conducted in the absence of any commercial or financial relationships that could be construed as a potential conflict of interest.

## Footnotes

1
https://github.com/smontazeriUH/Neonatal-EEGBackground-Classifier
