# Corrigendum: Building an open source classifier for the neonatal EEG background: a systematic feature-based approach from expert scoring to clinical visualization

**DOI:** 10.3389/fnhum.2024.1417744

**Published:** 2024-04-29

**Authors:** Saeed Montazeri, Elana Pinchefsky, Ilse Tse, Viviana Marchi, Jukka Kohonen, Minna Kauppila, Manu Airaksinen, Karoliina Tapani, Päivi Nevalainen, Cecil Hahn, Emily W. Y. Tam, Nathan J. Stevenson, Sampsa Vanhatalo

**Affiliations:** ^1^BABA Center, Pediatric Research Centre, Department of Clinical Neurophysiology, Children's Hospital and HUS Diagnostic Center, Helsinki University Hospital and University of Helsinki, Helsinki, Finland; ^2^Division of Neurology, Department of Paediatrics, Sainte-Justine University Hospital Centre, University of Montreal, Montreal, QC, Canada; ^3^Department of Developmental Neuroscience, Stella Maris Scientific Institute, IRCCS Fondazione Stella Maris Foundation, Pisa, Italy; ^4^Department of Computer Science, Aalto University, Espoo, Finland; ^5^Department of Signal Processing and Acoustics, Aalto University, Espoo, Finland; ^6^Department of Paediatrics (Neurology), The Hospital for Sick Children and University of Toronto, Toronto, ON, Canada; ^7^Brain Modelling Group, QIMR Berghofer Medical Research Institute, Brisbane, QLD, Australia; ^8^Neuroscience Center, Helsinki Institute of Life Science, University of Helsinki, Helsinki, Finland

**Keywords:** neonatal EEG, EEG monitoring, neonatal intensive care unit, background classifier, support vector machine, artificial neural network, EEG trend

In the published article, an author name was incorrectly written as “Saeed Montazeri Moghadam.” The correct spelling is “Saeed Montazeri.”

The authors apologize for this error and state that this does not change the scientific conclusions of the article in any way. The original article has been updated.

