# Supplementary material for: Building an Open Source Classifier for the Neonatal EEG Background: A Systematic Feature-Based Approach From Expert Scoring to Clinical Visualization
Source: Front Hum Neurosci. 2021 May 31;15:675154. doi: 10.3389/fnhum.2021.675154 (PMC8200402; doi:10.3389/fnhum.2021.675154)
Supplement: Supplementary file 1 [file Data_Sheet_1.PDF]

Supplementary Material

1 Design of neural network classifiers

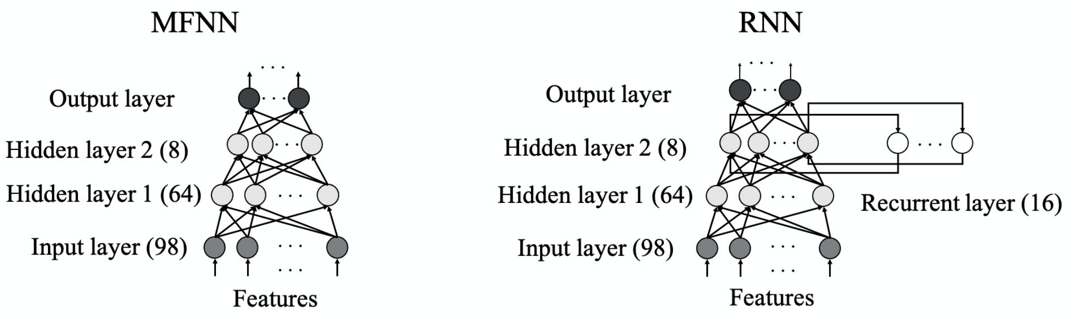

Figure S1. The architecture of the proposed four-layer feed-forward artificial neural network (MFNN; left) and the proposed recurrent neural network (RNN; right). The number of neurons in each layer is indicated in round brackets at the end of the layer's name. The number of neurons in the output layer changes with different classification approaches.

2 Flat vs. hierarchical/ SVM vs. MFNN vs. RNN/ SMOTE

The results comparing different classifier approaches for the 7-score classification problem are summarized in Tables S1. SMOTE decreased the performance of the classifiers. Since the RNN requires ordered sequential inputs and SMOTE disrupts the temporal sequence of epochs, this method is only applied to SVM and MFNN classifiers. Results show no considerable differences in performance between the flat approach and any of the hierarchical approaches (SVM - Flat $CI_{95\%}$: 87.1-87.6; Continuity $CI_{95\%}$: 86.8-87.2; Severity $CI_{95\%}$: 86.4-87.1). Comparison between flat approaches showed that RNN slightly outperforms SVM and MFNN classifiers (RNN $CI_{95\%}$: 88.6-89.1; MFNN $CI_{95\%}$: 87.3-87.9; SVM $CI_{95\%}$: 87.1-87.6).

Table S1. Classification performance averaged across all the test sets in LOSO cross-validation. Each classifier design can be trained by flat, **F**, or one of the hierarchical approaches (severity, **S**, or continuity, **C**). Distribution of scores in training data can be balanced by SMOTE algorithm, **Y** (yes), or **N** (no). All the classifiers are trained and tested on scores of E1 or E2 alone and the average performance is shown.

Classifier	Training			Performance					
	SMOTE (Y/N)	Flat / Hierarchical (F/S/C)	Training data	Test data	Avg. Acc (%)	Avg. F1 score (%)	Avg. W-Acc (%)	Avg. W-F1 score (%)	Avg. κ
SVM	N	F	E1/E2	E1/E2	87.4	44.7	81.3	56.2	0.42

	N	C	E1/E2	E1/E2	87.0	45.1	81.0	55.9	0.41
	N	S	E1/E2	E1/E2	87.2	45.1	81.0	55.7	0.41
	Y	F	E1/E2	E1/E2	85.3	43.0	78.1	53.3	0.37
	Y	C	E1/E2	E1/E2	86.2	42.9	77.4	53.3	0.37
	Y	S	E1/E2	E1/E2	86.5	43.1	77.0	54.1	0.38
MFNN	N	F	E1/E2	E1/E2	87.5	43.4	81.3	57.3	0.42
	N	C	E1/E2	E1/E2	86.4	43.7	79.8	54.0	0.39
	N	S	E1/E2	E1/E2	87.1	**45.4**	80.8	55.7	0.41
	Y	F	E1/E2	E1/E2	86.1	43.4	78.7	53.1	0.38
	Y	C	E1/E2	E1/E2	85.4	43.3	78.8	53.5	0.38
	Y	S	E1/E2	E1/E2	86.2	43.1	79.1	54.1	0.39
RNN	N	F	E1/E2	E1/E2	**88.8**	44.8	**82.7**	**60.1**	**0.43**

3 CONS vs. ALL annotations and post-processing

For benchmark, we compared the classifiers based on both *CONS* and *ALL* annotations with the 7-score scoring system in Figure S3A. Results of these two training approaches are compared to the mean RNN performance obtained in section 3.2.1 (green horizontal line in Figure S3A). A closer inspection of confusion matrices for both the *CONS* and *ALL* annotations shows essentially similar patterns in spread across disagreements: The scores tend to be confused with neighbouring scores only (Figure S3B&C). Moreover, the results showed that the performance of SVM and MFNN classifiers were mostly comparable. However, the RNN classifier was generally poorer.

Figure S3D demonstrates the time courses of annotation for one infant. Notice sharp peaks in the original signal lasting for one to few epochs. These are likely scoring/classification noise, due to ambiguity in the signal itself near the borderline between two scores rather than genuine change in brain state. Therefore, the annotations and classifier outputs were smoothed with a moving median window. For the annotations, we found that using a 5 epochs window length increased the inter-rater agreement (κ = 0.41 to 0.44) in the 7-score scoring system.

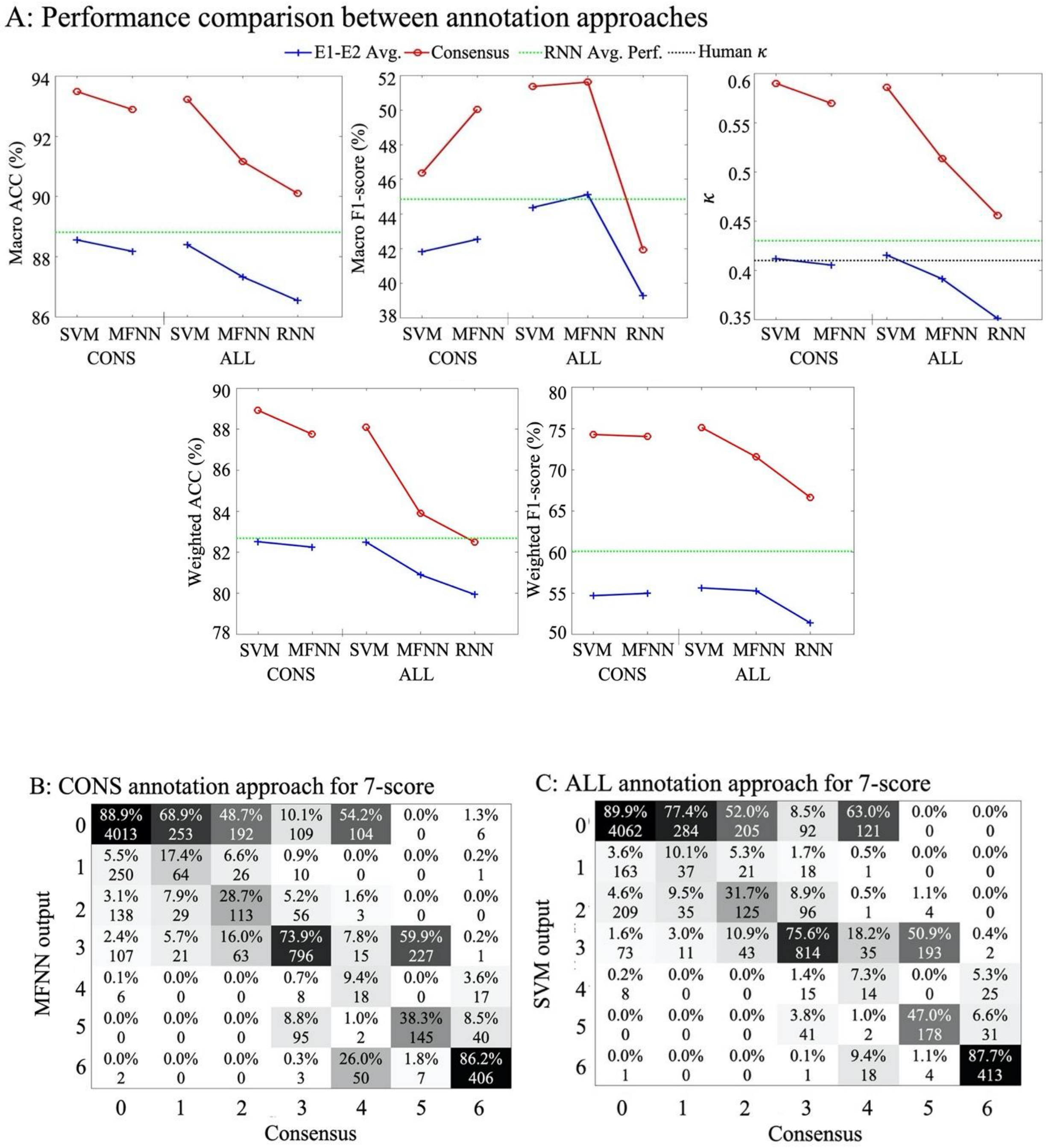
A: Performance comparison between annotation approaches
E1-E2 Avg.
Consensus
RNN Avg. Perf.
Human κ
Macro ACC (%)
Macro F1-score (%)
κ
Weighted ACC (%)
Weighted F1-score (%)
SVM MFNN SVM MFNN RNN
CONS ALL
B: CONS annotation approach for 7-score
MFNN output
Consensus
0: 88.9% 4013 | 68.9% 253 | 48.7% 192 | 10.1% 109 | 54.2% 104 | 0.0% 0 | 1.3% 6
1: 5.5% 250 | 17.4% 64 | 6.6% 26 | 0.9% 10 | 0.0% 0 | 0.0% 0 | 0.2% 1
2: 3.1% 138 | 7.9% 29 | 28.7% 113 | 5.2% 56 | 1.6% 3 | 0.0% 0 | 0.0% 0
3: 2.4% 107 | 5.7% 21 | 16.0% 63 | 73.9% 796 | 7.8% 15 | 59.9% 227 | 0.2% 1
4: 0.1% 6 | 0.0% 0 | 0.0% 0 | 0.7% 8 | 9.4% 18 | 0.0% 0 | 3.6% 17
5: 0.0% 0 | 0.0% 0 | 0.0% 0 | 8.8% 95 | 1.0% 2 | 38.3% 145 | 8.5% 40
6: 0.0% 2 | 0.0% 0 | 0.0% 0 | 0.3% 3 | 26.0% 50 | 1.8% 7 | 86.2% 406
C: ALL annotation approach for 7-score
SVM output
Consensus
0: 89.9% 4062 | 77.4% 284 | 52.0% 205 | 8.5% 92 | 63.0% 121 | 0.0% 0 | 0.0% 0
1: 3.6% 163 | 10.1% 37 | 5.3% 21 | 1.7% 18 | 0.5% 1 | 0.0% 0 | 0.0% 0
2: 4.6% 209 | 9.5% 35 | 31.7% 125 | 8.9% 96 | 0.5% 1 | 1.1% 4 | 0.0% 0
3: 1.6% 73 | 3.0% 11 | 10.9% 43 | 75.6% 814 | 18.2% 35 | 50.9% 193 | 0.4% 2
4: 0.2% 8 | 0.0% 0 | 0.0% 0 | 1.4% 15 | 7.3% 14 | 0.0% 0 | 5.3% 25
5: 0.0% 0 | 0.0% 0 | 0.0% 0 | 3.8% 41 | 1.0% 2 | 47.0% 178 | 6.6% 31
6: 0.0% 1 | 0.0% 0 | 0.0% 0 | 0.1% 1 | 9.4% 18 | 1.1% 4 | 87.7% 413

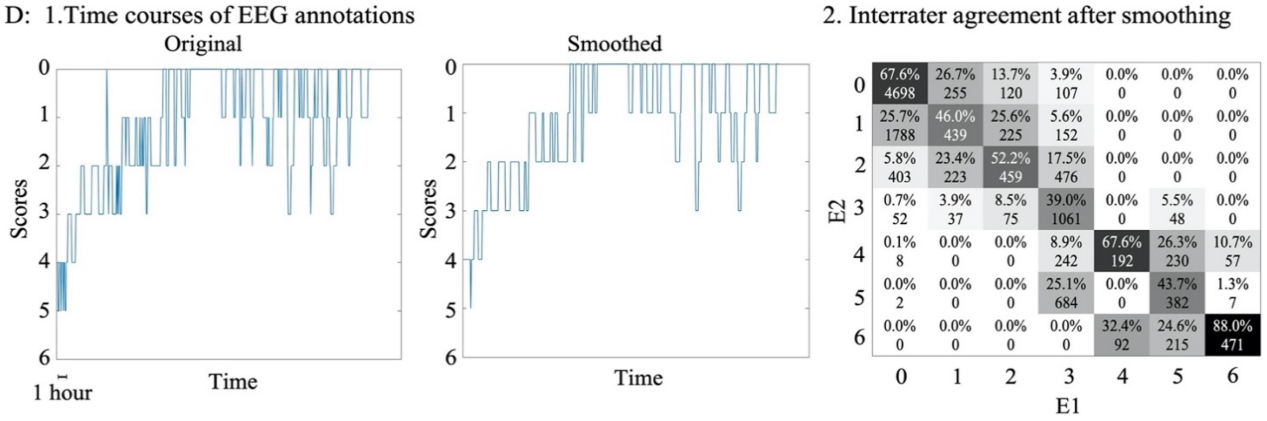

Figure S2. The CONS and ALL annotation approaches and effects of post-processing in 7-score scoring systems. (**A**) Performance comparison between classifiers. All results comparing classifiers to consensus epochs are shown with “o”, and the results comparison to average of E1 and E2 are displayed with “+”. The green dotted lines are averaged performance results for the RNN classifier when each classifier is trained on the scores of each annotator individually. The black dotted line is the inter-rater agreement between human experts (κ = 0.41). These lines are presented to provide a pairwise comparison between the results of the classifiers when trained to represent both annotators and each annotator individually. (**B**) Confusion matrix for MFNN classifier of the “CONS” annotation approach. (**C**) Confusion matrix for SVM classifier of the “ALL” annotation approach. (**D**) 1. Comparison of the time courses of EEG annotations in one subject before smoothing (left) and after smoothing (right) shows how random-appearing jumps in the score are effectively removed (moving median of 5 epochs). 2. Confusion matrices between experts for 7-score scores after smoothing. Note the increase in agreement as compared to Figure 2.

4 The optimal classifier

Performance results for classifiers trained with smoothed 5-score, flat approach, and without SMOTE are shown in Table S2. The results from the SVM, MFNN and RNN classifiers based on *ALL* -approach were mostly comparable, and generally better than classifiers trained with *CONS* annotations only.

Table S2. Classification performance. Each classifier is trained with smoothed 5-score, flat approach, and without SMOTE.

Classifier	Training data	Performance					
		Test data	Acc (%) ($CI_{95\%}$)	F1 score (%)	W-Acc (%)	W-F1 score (%)	κ ($CI_{95\%}$)
SVM	CONS	Consensus	96.7	57.5	95.6	90.4	0.75

			(95.4-97.8)				(0.73-0.76)
MFNN	CONS	Consensus	96.7 (95.1-97.8)	60.6	96.0	90.6	0.75 (0.72-0.76)
SVM	ALL	Consensus	97.1 (95.9-98.2)	70.8	95.9	92.2	0.78 (0.77-0.78)
MFNN	ALL	Consensus	97.0 (95.5-98.1)	69.3	95.5	91.8	0.76 (0.75-0.77)
RNN	ALL	Consensus	96.9 (95.5-98.0)	69.2	95.4	91.5	0.76 (0.75-0.77)

5 Dependency of kappa on the level of ambiguity

Each point in Figure S4 represents a relative error produced by one expert and the classifier against a reference (another expert) for each test subject. In (**A**) reference is E1 scores and in (**B**) reference is E2 scores. The lines drawn for each plot represent a fit of the error data with rho values (Pearson's linear correlation coefficient (***ρ***)) depicted in the plot. This infant level analysis shows a significant positive correlation, i.e. higher disagreements between human experts is associated with a higher disagreement between either human expert and the classifier.

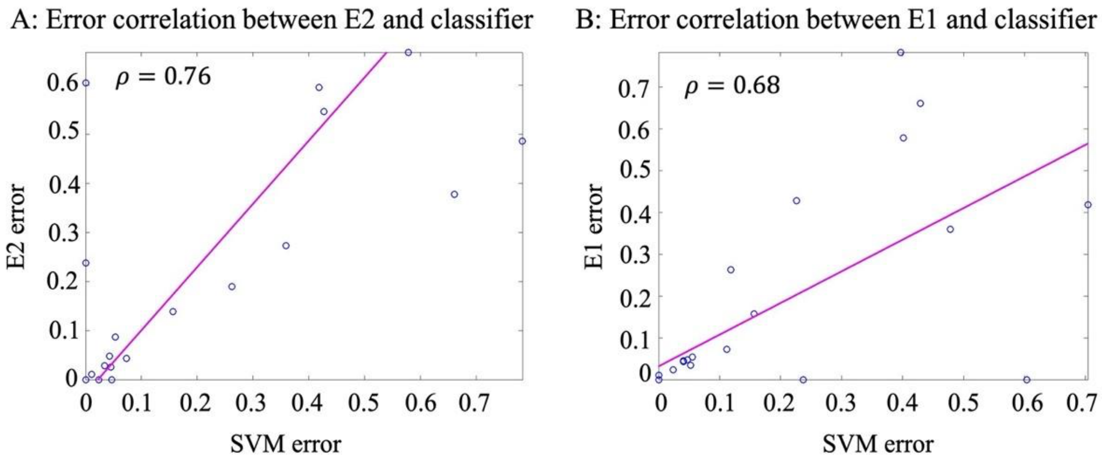

Figure S3. Classification error correlation plots. (**A**) when E1 is considered as reference scores and (**B**) when E2 is considered as reference scores. The lines drawn for each plot represent a fit of the error data and values written in each plot represent Pearson's linear correlation coefficient (***ρ***).

6 List of calculated features and results of the feature selection

The total 98 extracted features are listed in Table S3. Features are ordered based on the GA-based feature selection results under different levels of artefact thresholds. They are also categorized according to the domain of which they are computed (Type). All the features are computed per single channel except for Brain symmetry index (BSI) and Asymmetry index (ASI) which operate on at least one pair of channels. All feature algorithms are available in Github https://github.com/smontazeriUH/Neonatal-EEGBackground-Classifier.

Table S3. Feature set and feature evaluation results. Features are ordered by the average of selection frequencies over the four thresholds of the artefact rejection (0%, 10%, 25% and 50%). Notes: Type - indicates the domain of which feature is computed such as amplitude (A), information (I) and frequency (F).

#	Type	Feature description	Thresholds			
			0%	10%	25%	50%
1	A	Standard deviation of the amplitude modulation (µV) (Stevenson et al., 2013)	85	59	98	98
2	I	Intercept of linear fit to asymmetry vs burst duration (Iyer et al., 2014)	69	68	74	92
3	I	Multiscale Entropy (the average slope of the curve) (De Wel et al., 2017)	79	26	91	86
4	I	Multiscale Entropy (delta, 5 scales) (De Wel et al., 2017)	90	95	12	76
5	I	Detrended fluctuation analysis identifies the monofractal structures of the signal epoch (Peng et al., 1994; Kantelhardt et al., 2001)	53	65	64	73
6	I	Maximum value of the q-order singularity dimension curve. (Ihlen, 2012)	55	63	62	73
7	A	Mean of the amplitude modulation (µV) (Stevenson et al., 2013)	68	0	94	80

8	F	Power in frequency sub-band (10 - 12 Hz) (Temko et al., 2011)	67	66	52	49
9	I	Non-linear energy (Temko et al., 2011)	51	45	63	72
10	I	Hjorth 2nd derivatives (Temko et al., 2011)	63	69	64	30
11	A	Number of bursts (Palmu et al., 2010)	53	52	53	67
12	I	4th order autoregressive modelling error (Temko et al., 2011)	44	50	75	54
13	I	2nd order autoregressive modelling error (Temko et al., 2011)	40	52	71	59
14	F	Brain symmetry index (van Putten, 2007)	38	75	64	45
15	A	Skewness of the amplitude modulation (Stevenson et al., 2013)	59	75	33	53
16	F	Normalized power in frequency sub-band (12 - 30 Hz) (Temko et al., 2011)	60	60	40	60
17	I	3rd order autoregressive modelling error (Temko et al., 2011)	58	66	66	19
18	F	Power in frequency sub-band (9 - 11 Hz) (Temko et al., 2011)	65	57	31	56
19	I	Alpha (truncated power law fit to CDF of burst size) (Iyer et al., 2014)	31	44	58	76
20	F	Power in frequency sub-band (6 - 8 Hz) (Temko et al., 2011)	69	0	82	54

21	F	Spectral Edge Frequency from (80%) (Temko et al., 2011)	54	26	58	67
22	I	Covariance between instantaneous amplitude and instantaneous frequency describes the relationship between the amplitude and frequency modulation (µV*Hz) (Stevenson et al., 2013)	0	66	66	71
23	F	Power in frequency sub-band (5 - 7 Hz) (Temko et al., 2011)	63	72	27	39
24	I	Multifractal detrended fluctuation analysis (Ihlen, 2012)	35	45	61	54
25	A	Inter-burst interval (sec) (Palmu et al., 2010)	0	64	65	64
26	I	1st order autoregressive modelling error (Temko et al., 2011)	59	44	65	20
27	F	Power in frequency sub-band (12 - 30 Hz) (Temko et al., 2011)	0	78	47	61
28	F	Power in frequency sub-band (8 - 10 Hz) (Temko et al., 2011)	19	61	44	59
29	F	Power in frequency sub-band (9 - 11 Hz) (Temko et al., 2011)	59	15	86	22
30	A	Variance first derivative (Temko et al., 2011)	55	38	31	58
31	I	Log-Likelihood Ratio of Fit (truncated power law fit to CDF of burst duration) (Iyer et al., 2014)	17	54	63	48
32	I	5th order autoregressive modelling error (Temko et al., 2011)	40	44	56	40

33	A	Averaged skewness over bursts with a duration of 62-125ms (Iyer et al., 2015)	60	43	56	21
34	F	Normalized power in frequency sub-band (8 - 10 Hz) (Temko et al., 2011)	39	47	35	54
35	I	Slope of linear fit to asymmetry vs burst duration (Iyer et al., 2014)	67	64	14	30
36	I	Log-Likelihood Ratio of Fit (truncated power law fit to CDF of burst area) (Iyer et al., 2014)	3	52	61	58
37	F	Activation synchrony index (Rasanen et al., 2013)	48	4	97	22
38	F	Peak frequency of spectrum (Temko et al., 2011)	53	0	66	51
39	I	Multiscale Entropy (max) (De Wel et al., 2017)	55	0	66	48
40	I	8th order autoregressive modelling error (Temko et al., 2011)	40	29	76	23
41	A	Kurtosis of the amplitude modulation (Stevenson et al., 2013)	28	0	80	59
42	A	Kurtosis (Temko et al., 2011)	56	29	32	50
43	A	Mean burst duration (Iyer et al., 2014)	21	69	57	20
44	A	Difference between the 95th and 5th percentiles of peak-to-peak amplitude (rEEG; range-EEG) (µV) (Navakatikyan et al., 2016)	44	55	48	19
45	A	Duration of bursts (sec) (Palmu et al., 2010)	58	57	36	15
46	F	Total power (0-30 Hz) (Temko et al., 2011)	19	12	67	68

47	A	Averaged kurtosis over bursts with a duration of 62-125ms (Iyer et al., 2015)	60	47	41	18
48	I	Fisher information (Temko et al., 2011)	54	0	49	62
49	A	Averaged skewness over bursts with a duration of 250-500ms (Iyer et al., 2015)	57	0	55	53
50	I	Shannon entropy (Greene et al., 2008; Temko et al., 2011)	54	65	0	45
51	I	Suppression curve (Dereymaeker et al., 2016)	0	44	84	34
52	F	Spectral Edge Frequency from (90%) (Temko et al., 2011)	55	0	37	67
53	F	Power in frequency sub-band (7 - 9 Hz) (Temko et al., 2011)	0	59	79	20
54	F	Normalized power in frequency sub-band (4 - 6 Hz) (Temko et al., 2011)	57	0	67	34
55	F	Mean instantaneous frequency of the epoch (Hz) (Stevenson et al., 2013)	58	0	79	19
56	F	Kurtosis instantaneous frequency of the epoch (Stevenson et al., 2013)	0	68	68	17
57	I	Higuchi fractal dimension (Higuchi, 1988)	72	0	59	18
58	I	Log-Likelihood Ratio of Fit (truncated power law fit to CDF of burst area) (Iyer et al., 2014)	33	37	58	20
59	A	Coefficient of variation (burst durations) (Iyer et al., 2014)	36	15	72	24

60	I	Lambda (truncated power law fit to CDF of burst duration) (Iyer et al., 2014)	9	55	62	18
61	F	Normalized power in frequency sub-band (5 - 7 Hz) (Temko et al., 2011)	49	0	73	21
62	I	6th order autoregressive modelling error (Temko et al., 2011)	57	43	30	12
63	F	Power in frequency sub-band (1 - 3 Hz) (Temko et al., 2011)	35	0	62	45
64	F	Normalized power in frequency sub-band (3 - 5 Hz) (Temko et al., 2011)	38	0	77	25
65	F	Skewness instantaneous frequency of the epoch (Stevenson et al., 2013)	0	0	76	60
66	A	Averaged skewness over bursts with a duration of 125-250ms (Iyer et al., 2015)	43	59	22	11
67	I	Spectral entropy (Temko et al., 2011)	55	0	21	58
68	I	Singular value decomposition entropy (Temko et al., 2011)	47	17	25	42
69	F	Power in frequency sub-band (4 - 6 Hz) (Temko et al., 2011)	24	47	45	13
70	F	Power in frequency sub-band (2 - 4 Hz) (Temko et al., 2011)	68	0	42	14
71	F	Normalized power in frequency sub-band (2 - 4 Hz) (Temko et al., 2011)	33	0	72	19

72	I	Alpha (truncated power law fit to CDF of burst duration) (Iyer et al., 2014)	23	23	59	18
73	F	Normalized power in frequency sub-band (0 - 2 Hz) (Temko et al., 2011)	64	0	47	11
74	F	Power in frequency sub-band (3 - 5 Hz) (Temko et al., 2011)	38	0	63	19
75	A	Zero crossings (Temko et al., 2011)	33	0	68	18
76	F	Variance instantaneous frequency of the epoch (Hz) (Stevenson et al., 2013)	0	22	65	29
77	I	Hjorth parameter (variance) (Temko et al., 2011)	65	0	36	15
78	F	Wavelet energy (average of absolute value of eight coefficients using the Daubechy four wavelet) (Temko et al., 2011)	0	54	44	16
79	A	Skewness (Temko et al., 2011)	49	0	48	16
80	F	Spectral Edge Frequency from (95%) (Temko et al., 2011)	26	69	12	4
81	A	Root mean squared amplitude (Temko et al., 2011)	61	0	4	41
82	F	Power in frequency sub-band (0 - 2 Hz) (Temko et al., 2011)	4	0	35	65
83	I	7th order autoregressive modelling error (Temko et al., 2011)	0	7	77	19
84	A	Averaged kurtosis over bursts with a duration of 250-500ms (Iyer et al., 2015)	20	0	56	25

85	F	Normalized power in frequency sub-band (1 - 3 Hz) (Temko et al., 2011)	0	0	78	19
86	I	s_{min} (truncated power law fit to CDF of burst area) (Iyer et al., 2014)	7	4	58	19
87	F	Normalized power in frequency sub-band (7 - 9 Hz) (Temko et al., 2011)	0	26	13	45
88	I	9th order autoregressive modelling error (Temko et al., 2011)	0	39	30	11
89	I	Hjorth 1st derivatives (Temko et al., 2011)	40	0	0	39
90	I	Variance second derivative (Temko et al., 2011)	0	0	55	19
91	A	Median of rEEG (µV) (Navakatikyan et al., 2016)	0	37	2	30
92	A	Number of minima and maxima (Temko et al., 2011)	0	0	52	16
93	I	Zero crossings second derivative (Temko et al., 2011)	0	38	0	30
94	I	s_{min} (truncated power law fit to CDF of burst duration) (Iyer et al., 2014)	14	36	2	4
95	F	Normalized power in frequency sub-band (6 - 8 Hz) (Temko et al., 2011)	0	0	40	12
96	A	Averaged kurtosis over bursts with a duration of 125-250ms (Iyer et al., 2015)	0	0	37	15
97	I	Line-length (sum of the absolute differences between all consecutive samples) (Koolen et al., 2014)	23	0	0	1

98	F	Normalized power in frequency sub-band (10 - 12 Hz) (Temko et al., 2011)	0	0	0	0

7 References

De Wel, O., Lavanga, M., Dorado, A.C., Jansen, K., Dereymaeker, A., Naulaers, G., et al. (2017). Complexity analysis of neonatal EEG using multiscale entropy: applications in brain maturation and sleep stage classification. *Entropy* 19(10)**,** 516. doi: 10.3390/e19100516.

Dereymaeker, A., Koolen, N., Jansen, K., Vervisch, J., Ortibus, E., De Vos, M., et al. (2016). The suppression curve as a quantitative approach for measuring brain maturation in preterm infants. *Clin Neurophysiol* 127(8)**,** 2760-2765. doi: 10.1016/j.clinph.2016.05.362.

Greene, B.R., Faul, S., Marnane, W.P., Lightbody, G., Korotchikova, I., and Boylan, G.B. (2008). A comparison of quantitative EEG features for neonatal seizure detection. *Clin Neurophysiol* 119(6)**,** 1248-1261. doi: 10.1016/j.clinph.2008.02.001.

Higuchi, T. (1988). Approach to an irregular time series on the basis of the fractal theory. *Physica D* 31(2)**,** 277-283. doi: 10.1016/0167-2789(88)90081-4.

Ihlen, E.A. (2012). Introduction to multifractal detrended fluctuation analysis in matlab. *Front Physiol* 3**,** 141. doi: 10.3389/fphys.2012.00141.

Iyer, K.K., Roberts, J.A., Hellstrom-Westas, L., Wikstrom, S., Hansen Pupp, I., Ley, D., et al. (2015). Cortical burst dynamics predict clinical outcome early in extremely preterm infants. *Brain* 138(Pt 8)**,** 2206-2218. doi: 10.1093/brain/awv129.

Iyer, K.K., Roberts, J.A., Metsaranta, M., Finnigan, S., Breakspear, M., and Vanhatalo, S. (2014). Novel features of early burst suppression predict outcome after birth asphyxia. *Ann Clin Transl Neurol* 1(3)**,** 209-214. doi: 10.1002/acn3.32.

Kantelhardt, J.W., Koscielny-Bunde, E., Rego, H.H., Havlin, S., and Bunde, A. (2001). Detecting long-range correlations with detrended fluctuation analysis. *Physica A* 295(3-4)**,** 441-454. doi: 10.1016/S0378-4371(01)00144-3.

Koolen, N., Jansen, K., Vervisch, J., Matic, V., De Vos, M., Naulaers, G., et al. (2014). Line length as a robust method to detect high-activity events: automated burst detection in premature EEG recordings. *Clin Neurophysiol* 125(10)**,** 1985-1994. doi: 10.1016/j.clinph.2014.02.015.

Navakatikyan, M.A., O'Reilly, D., and Van Marter, L.J. (2016). Automatic measurement of interburst interval in premature neonates using range EEG. *Clin Neurophysiol* 127(2)**,** 1233-1246. doi: 10.1016/j.clinph.2015.11.008.

Palmu, K., Stevenson, N., Wikstrom, S., Hellstrom-Westas, L., Vanhatalo, S., and Palva, J.M. (2010). Optimization of an NLEO-based algorithm for automated detection of spontaneous activity transients in early preterm EEG. *Physiol Meas* 31(11)**,** N85-93. doi: 10.1088/0967-3334/31/11/N02.

Peng, C.K., Buldyrev, S.V., Havlin, S., Simons, M., Stanley, H.E., and Goldberger, A.L. (1994). Mosaic organization of DNA nucleotides. *Phys Rev E Stat Phys Plasmas Fluids Relat Interdiscip Topics* 49(2)**,** 1685-1689. doi: 10.1103/physreve.49.1685.

Rasanen, O., Metsaranta, M., and Vanhatalo, S. (2013). Development of a novel robust measure for interhemispheric synchrony in the neonatal EEG: activation synchrony index (ASI). *Neuroimage* 69**,** 256-266. doi: 10.1016/j.neuroimage.2012.12.017.

Stevenson, N.J., Korotchikova, I., Temko, A., Lightbody, G., Marnane, W.P., and Boylan, G.B. (2013). An automated system for grading EEG abnormality in term neonates with hypoxic-

ischaemic encephalopathy. *Ann Biomed Eng* 41(4), 775-785. doi: 10.1007/s10439-012-0710-5.
Temko, A., Thomas, E., Marnane, W., Lightbody, G., and Boylan, G. (2011). EEG-based neonatal seizure detection with Support Vector Machines. *Clin Neurophysiol* 122(3), 464-473. doi: 10.1016/j.clinph.2010.06.034.
van Putten, M.J. (2007). The revised brain symmetry index. *Clin Neurophysiol* 118(11), 2362-2367. doi: 10.1016/j.clinph.2007.07.019.
